# *LMO* family gene polymorphisms and Wilms tumor susceptibility in Chinese children: a five-center case-control study

**DOI:** 10.1186/s12885-024-12557-3

**Published:** 2024-06-27

**Authors:** Wen Fu, Linqing Deng, Xiaosong Yan, Rui-Xi Hua, Jiao Zhang, Haixia Zhou, Changmi Deng, Suhong Li, Jiwen Cheng, Jichen Ruan, Jing He, Guochang Liu

**Affiliations:** 1 Department of Pediatric Surgery, Guangzhou Institute of Pediatrics, Guangdong Provincial Key Laboratory of Research in Structural Birth Defect Disease, Guangdong Provincial Clinical Research Center for Child Health, Guangzhou Women and Children’s Medical Center, Guangzhou Medical University, 9 Jinsui Road, Guangzhou, Guangdong 510623 China; 2 Department of Pathology, The Affiliated Children’s Hospital of Xi’an Jiaotong University, Xi’an, Shaanxi 710003 China; 3 Department of Pediatric Surgery, The First Affiliated Hospital of Zhengzhou University, Zhengzhou, Henan, 450052 China; 4 Department of Hematology, The Key Laboratory of Pediatric Hematology and Oncology Diseases of Wenzhou, The Second Affiliated Hospital, Yuying Children’s Hospital of Wenzhou Medical University, Wenzhou, Zhejiang 325027 China; 5 Department of Pathology, Children Hospital and Women Health Center of Shanxi, Taiyuan, Shannxi 030013 China; 6 Department of Pediatric Surgery, The Second Affiliated Hospital of Xi’an Jiaotong University, Xi’an, Shaanxi 710004 China

**Keywords:** Wilms tumor, Susceptibility, LMO family genes, Polymorphism

## Abstract

**Background:**

Wilms tumor is the most prevalent embryonal kidney malignancy in children worldwide. Previous genome-wide association study (GWAS) identified that LIM domain only 1 (*LMO1*) gene polymorphisms affected the susceptibility to develop certain tumor types. Apart from *LMO1*, the *LMO* gene family members also include *LMO2-4*, each of which has oncogenic potential.

**Methods:**

We conducted this five-center case‒control study to assess the correlations between single nucleotide polymorphisms in *LMO* family genes and Wilms tumor susceptibility. Odds ratios and 95% confidence intervals were calculated to evaluate the strength of the association.

**Results:**

We found *LMO1* rs2168101 G > T and rs11603024 C > T as well as *LMO2* rs7933499 G > A were significantly associated with Wilms tumor risk. Stratified analysis demonstrated a protective role of rs2168101 GT/TT genotypes against Wilms tumor in the subgroups of age ≤ 18 months, males and clinical stages I/II compared to the rs2168101 GG genotype. Nevertheless, carriers with the rs11603024 TT genotype were more likely to have an increased risk of Wilms tumor than those with rs11603024 CC/CT genotypes in age > 18 months. And the rs11603024 was identified as a protective polymorphism for reducing the risk of Wilms tumor in the sex- and gender- subgroup. Likewise, carriers with the rs7933499 GA/AA genotypes were at significantly elevated risk of Wilms tumor in age ≤ 18 months and clinical stages I/II.

**Conclusion:**

Overall, our study identified the importance of *LMO* family gene polymorphisms on Wilms tumor susceptibility in Chinese children. Further investigations are needed to validate our conclusions.

**Graphical Abstract:**

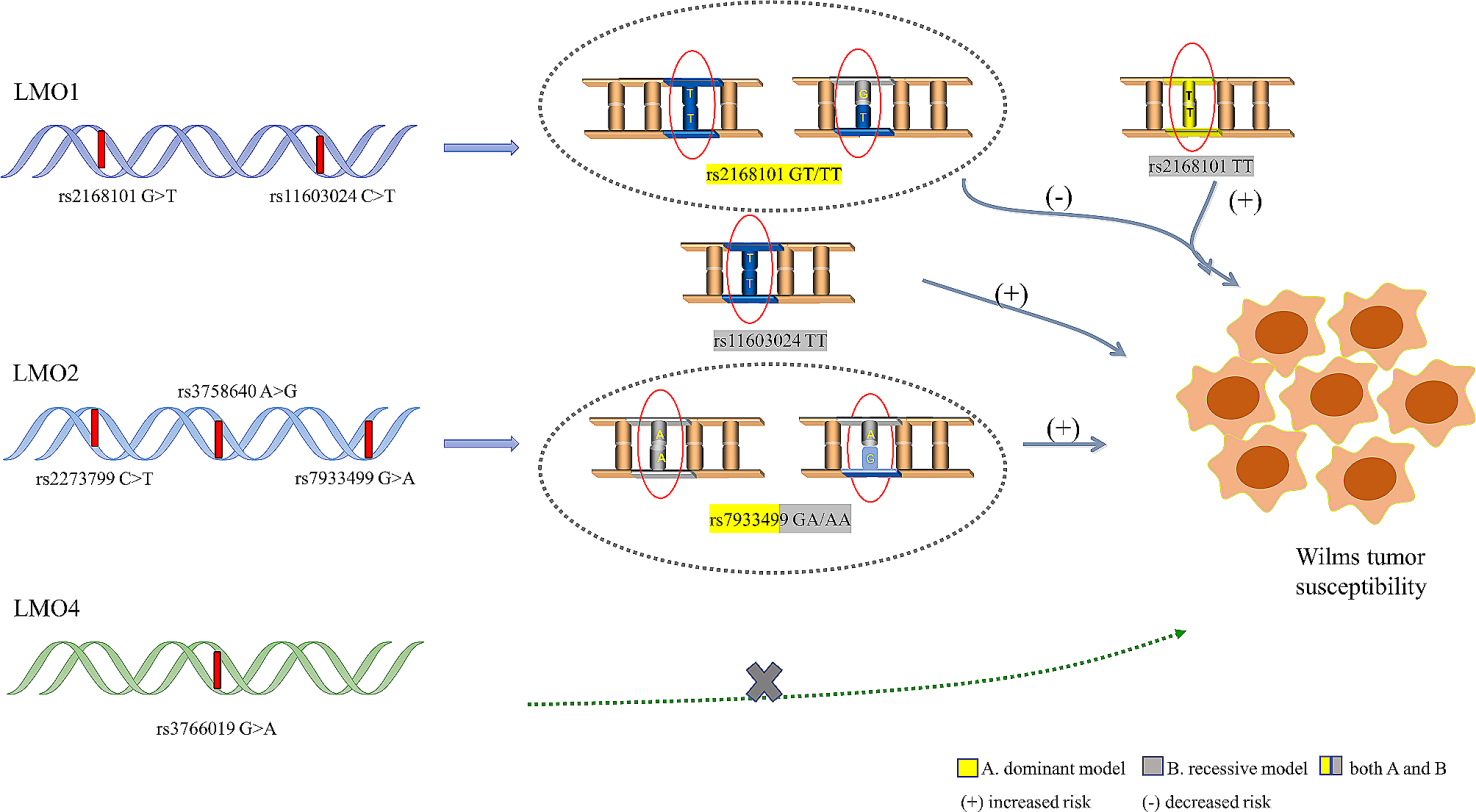

**Supplementary Information:**

The online version contains supplementary material available at 10.1186/s12885-024-12557-3.

## Introduction

Wilms tumor (WT), or nephroblastoma, is mainly derived from abnormal renal development, especially the failure of metanephric precursor differentiation, and has classified triphasic histology [1]. Wilms tumor is considered a common kidney malignancy affecting young children under 15 years old, accounting for nearly 90% of pediatric renal tumors and approximately 7% of childhood cancers [2, 3]. The incidence and distribution of WT varies widely by race and geography, with the highest incidence of 10 cases per million children in populations of Western black ancestry and the lowest incidence of 3–4 cases per million children in East Asian populations [4, 5]. Currently, with the exception of high-risk, histologically unfavorable, bilateral and recurrent cases, more than 90% of WT patients can benefit from improved modern multimodality therapy and refined risk stratification [6]. However, a proportion of survivors still suffer from chronic adverse health problems and social outcomes [6]. Unlike adult tumors, which are more influenced by the environment and unhealthy lifestyle, pediatric tumors appear to be more susceptible to genetic variation [7]. As published, the genetic landscape of Wilms tumor is diverse and involves approximately 40 cancer genes [8]. Early information on Wilms tumor-associated genes were identified in patients with tumor predisposition syndromes, highlighting the vital roles of Wilms tumor 1 (*WT1*), *WTX*, the WNT pathway and so on [8–10]. Subsequently, several novel cancer-related genes underpinning the occurrence of Wilms tumor have been primarily deciphered, including microRNA processing genes (*DROSHA, DICER1, DGCR8*, *XPO5* and *TARBP2*) [1, 8, 11, 12], transcription factors (*SIX1* and *SIX2*) [1, 13], and epigenetic remodelers (*SMARCA4* and *ARID1A*) [8, 14]. Despite significant efforts to study oncogenes in Wilms tumor, we must acknowledge that the genes identified to date could only explain a small part of the etiology of Wilms tumor.

A genome-wide association study (GWAS) is an extremely useful method to focus on the relationship between susceptible genes and complicated human diseases, including cancers [7, 15]. Previous GWAS and candidate gene approaches have discovered some susceptible genes correlated with the predisposition to Wilms tumor, such as *METTL3* [16], *ALKBH5* [17], *BRAD1* [18], *PHOX2B* [19], and *LMO1* [20]. The *LMO* gene family comprises four members, *LMO1-4*, which encode many transcriptional cofactors with a common structure of only two tandem LIM domains [21]. Accumulating evidence indicates that the *LMO* gene family is strongly linked to the occurrence and progression of several cancers [21]. For instance, *LMO1* and *LMO2* were first described in a chromosomal translocation event at different loci in T-cell leukemia [22]. *LMO1* and *LMO3* both contribute to the development of neuroblastoma [23, 24]. *LMO4* was originally found to be overexpressed in a proportion of breast cancer patients and related to poor prognosis [21, 25].

Currently, there are no genetic association studies between other members of the *LMO* family gene polymorphisms and Wilms tumor susceptibility. In addition to a replication study of the *LMO1* gene in a larger population, we also need further investigation to validate the associations between other underlying SNPs in the *LMO1* gene and Wilms tumor susceptibility. With this in mind, we carried out this five-center case‒control study to comprehensively explore whether SNPs in the *LMO* gene family influence Wilms tumor susceptibility in a larger Chinese population Our current study was the first to explore the associations and contributed to understanding the role of *LMO* familial genetic variants in the mechanisms of Wilms tumor tumorigenesis, thus filling a gap in this field.

## Materials and methods

### Study population

A total of 414 Wilms tumor-bearing patients and 1199 cancer-free controls were recruited from five independent hospitals in this case‒control study, as reported previously (Table S1) [17, 26]. All patients were confirmed to have a diagnosis of Wilms tumor by histopathology. Matched to the cases by age, sex and race, the controls were randomly selected from healthy volunteers who received a physical examination at the same hospitals during the periods.

### Genotyping

We collected approximately 2 ml of peripheral blood from each participant for DNA extraction and carefully screened for six potentially functional SNPs in the *LMO* family genes, including rs2168101 G > T and rs11603024 C > T in the *LMO1* gene, rs2273799 C > T, rs3758640 A > G and rs7933499 G > A in the *LMO2* gene, and rs3766019 G > A in the *LMO4* gene. The *LMO1* gene rs2168101 G > T was selected according to a previous publication [27], while the remaining five functional SNPs were chosen from the websites of the NCBI dbSNP database (http://www.ncbi.nlm.nih.gov/projects/SNP) and SNPinfo (http://snpinfo.niehs.nih.gov/snpfunc.html). The selection criteria for screening candidate SNPs have been described previously [28, 29]. In brief, we selected functional SNPs located in the 5′ and 3′ untranslated region (UTR), 5′ flanking region, exons, and introns of the *LMO* family genes. The minor allele frequencies (MAFs) of the chosen SNPs were at or above 5% in the Chinese Han population. Moreover, the linkage disequilibrium (LD) between selected SNPs was low (R^2^ < 0.8). We included other new *LMO1*, *LMO2* and *LMO4* SNPs with potential functions. The rs11603024 in the *LMO1* gene, rs2273799 and rs3758640 in the *LMO2* gene may affect the function of transcription factor binding site (TFBS), while *LMO2* rs7933499 might affect the binding capacity of miRNA and the splicing process (Table S2). DNA was extracted from each subject’s peripheral blood sample using the Genomic DNA kit (Tian Gen Biotech Co. Ltd., Beijing, China), and genotyping of the *LMO* family gene polymorphisms was performed by the TaqMan real-time PCR assay. In addition, we randomly selected 10% of all samples for repeated assays to avoid the incorrect experimental results, and the results of regenotyping were in 100% concordance with the original results.

### Statistical analysis

We then assessed the concordance of genotype frequencies with Hardy-Weinberg Equilibrium (HWE) for the control group through a χ^2^ goodness-of-fit test. A two-sided χ^2^ test was used to detect the differences in genotype frequencies and demographic variables between cases and healthy controls. To further investigate the relationship between the *LMO* family gene polymorphisms and Wilms tumor risk, we also applied unconditional logistic regression to calculate age- and sex-adjusted odds ratios (ORs), 95% confidence intervals (CIs), and two-sided *P* values. Besides, the Genotype-Tissue Expression (GTEx) (https://gtexportal.org) was applied to performing the expression quantitative trait locus (eQTL) analysis, assessing whether significant SNPs affecting the expression level of corresponding genes or nearby genes. The results were considered statistically significant if *P* < 0.05. All statistical analyses were performed rigorously with SAS 9.1 software (SAS Institute, Cary, NC, USA).

## Results

### Association between *LMO* family gene polymorphisms and Wilms tumor susceptibility

According to the current study, 403 cases and 1198 cancer-free controls were successfully genotyped for the six SNPs in the *LMO* family gene out of a total of 414 cases and 1199 controls. The correlation of *LMO1* gene, *LMO2* gene and *LMO4* gene polymorphisms with Wilms tumor susceptibility is described in detail in Table 1. The genotype frequencies of the control groups were all consistent with HWE (*P* = 0.367 for rs2168101 G > T, *P* = 0.487 for rs11603024 C > T, *P* = 0.639 for rs2273799 C > T, *P* = 0.712 for rs3758640 A > G, *P* = 0.929 for rs7933499 G > A, and *P* = 0.599 for rs3766019 G > A). Regarding the *LMO1* gene, rs2168101 G > T polymorphism was associated with Wilms tumor susceptibility. Specifically, the *LMO1* rs2168101 GT/TT genotypes were found to decrease the risk of developing Wilms tumor in the dominant model (adjusted OR = 0.74, 95% CI = 0.59–0.93, *P* = 0.009). In contrast, subjects carrying the *LMO1* rs2168101 TT genotype might have an increased risk of Wilms tumor in the recessive model (adjusted OR = 1.79, 95% CI = 1.25–2.57, *P* = 0.001). Moreover, another SNP in the *LMO1* gene, rs11603024 C > T, was detected to increase Wilms tumor risk in a recessive model (adjusted OR = 4.29, 95% CI = 2.08–8.84, *P* < 0.0001). Regarding the *LMO2* gene, among the three genotyped SNPs (rs2273799, rs3758640, and rs7933499), only the rs7933499 G > A was significantly associated with an increased risk of developing Wilms tumor (dominant model: adjusted OR = 1.37, 95% CI = 1.03–1.82, *P* = 0.032 and recessive model: adjusted OR = 4.91, 95% CI = 2.11–11.46, *P* = 0.0002). For the *LMO4* gene, there were no obviously significant correlations between the rs3766019 G > A polymorphism and Wilms tumor susceptibility.

**Table 1:** Association between polymorphisms in *LMO* family genes with Wilms tumor susceptibility

Gene	Polymorphism	Allele		Case			Control			AOR (95% CI) ^a^	*P* ^a^	AOR (95% CI) ^b^	*P* ^b^	HWE
		A	B	AA	AB	BB	AA	AB	BB					
*LMO1*	rs2168101	G	T	227	127	53	587	501	94	**0.74 (0.59–0.93)**	**0.009**	**1.79 (1.25–2.57)**	**0.001**	0.367
*LMO1*	rs11603024	C	T	307	72	18	925	244	13	1.06 (0.81–1.39)	0.691	**4.29 (2.08–8.84)**	**< 0.0001**	0.487
*LMO2*	rs2273799	C	T	144	177	70	383	586	212	0.83 (0.65–1.05)	0.126	1.01 (0.75–1.36)	0.974	0.639
*LMO2*	rs3758640	A	G	170	169	52	518	524	139	1.03 (0.81–1.29)	0.833	1.17 (0.83–1.64)	0.379	0.712
*LMO2*	rs7933499	G	A	306	71	14	981	191	9	**1.37 (1.03–1.82)**	**0.032**	**4.91 (2.11–11.46)**	**0.0002**	0.929
*LMO4*	rs3766019	G	A	277	110	4	814	336	31	0.91 (0.71–1.17)	0.475	0.38 (0.14–1.09)	0.073	0.599

AOR, adjusted odds ratio; CI, confidence interval, HWE, Hardy**-**Weinberg equilibrium

^a^ Adjusted for age and sex for dominant model

^b^ Adjusted for age and sex for recessive model

### Stratification analysis

We then further performed a stratification analysis on some significant SNPs that were related to Wilms tumor by age, sex and clinical stage and explored the relationships between significant SNPs derived from *LMO* family genes and Wilms tumor risk. The obtained results are depicted in Table 2. We found that rs2168101 GT/TT genotypes played a protective role against the occurrence of Wilms tumor in the subgroups of age ≤ 18 months (adjusted OR = 0.59, 95% CI = 0.40–0.88, *P* = 0.008), males (adjusted OR = 0.69, 95% CI = 0.50–0.94, *P* = 0.020) and clinical stages I/II (adjusted OR = 0.74, 95% CI = 0.56–0.97, *P* = 0.031) compared to the rs2168101 GG genotype. Nevertheless, carriers with the rs11603024 TT genotype were significantly related to an increased risk of Wilms tumor than carriers with rs11603024 CC/CT genotypes among children aged over 18 months (adjusted OR = 5.49, 95% CI = 2.16–13.98, *P* = 0.0004), females (adjusted OR = 5.72, 95% CI = 1.70-19.25, *P* = 0.005), males (adjusted OR = 3.63, 95% CI = 1.45–9.07, *P* = 0.006), clinical stages I/II (adjusted OR = 2.67, 95% CI = 1.05–6.78, *P* = 0.040) and clinical stages III/IV (adjusted OR = 7.56, 95% CI = 3.23–17.67, *P* < 0.0001). Similarly, we found that carriers with rs7933499 GA/AA genotypes significantly increased the risk of Wilms tumor in cases aged ≤ 18 (adjusted OR = 1.74, 95% CI = 1.08–2.81, *P* = 0.023) and clinical stages I/II (adjusted OR = 1.49, 95% CI = 1.06–2.09, *P* = 0.021) compared to subjects with the rs7933499 GG genotype.

**Table 2:** Stratification analysis of genotypes derived from *LMO* family genes with Wilms tumor risk

Variables	rs2168101 (cases/controls)		AOR (95% CI) ^a^	*P* ^a^	rs11603024 (cases/controls)		AOR (95% CI) ^a^	*P* ^a^	rs7933499 (cases/controls)		AOR (95% CI) ^a^	*P* ^a^
	GG	GT/TT			CC/CT	TT			GG	GA/AA		
Age, month												
≤ 18	89/233	52/227	**0.59 (0.40–0.88)**	**0.008**	136/454	5/6	2.66 (0.80–8.88)	0.112	106/393	31/67	**1.74 (1.08–2.81)**	**0.023**
> 18	138/354	118/368	0.89 (0.60–1.06)	0.118	243/715	13/7	**5.49 (2.16–13.98)**	**0.0004**	200/588	54/133	1.20 (0.84–1.71)	0.315
Sex												
Females	101/247	86/268	0.78 (0.56–1.10)	0.155	179/511	8/4	**5.72 (1.70-19.25)**	**0.005**	144/426	40/88	1.35 (0.89–2.05)	0.166
Males	126/340	84/327	**0.69 (0.50–0.94)**	**0.020**	200/658	10/9	**3.63 (1.45–9.07)**	**0.006**	162/555	45/112	1.41 (0.95–2.08)	0.087
Clinical stages												
I/II	139/587	103/595	**0.74 (0.56–0.97)**	**0.031**	235/1169	7/13	**2.67 (1.05–6.78)**	**0.040**	184/981	55/200	**1.49 (1.06–2.09)**	**0.021**
III/IV	76/587	62/595	0.80 (0.56–1.14)	0.208	128/1169	10/13	**7.56 (3.23–17.67)**	**< 0.0001**	109/981	26/200	1.15 (0.73–1.81)	0.547

CI: confidence interval; AOR: adjusted odds ratio

^a^ Obtained in logistic regression models with adjustment for age and sex omitting the corresponding stratification factor

### The eQTL analysis of *LMO* family gene

**Fig. 1:**
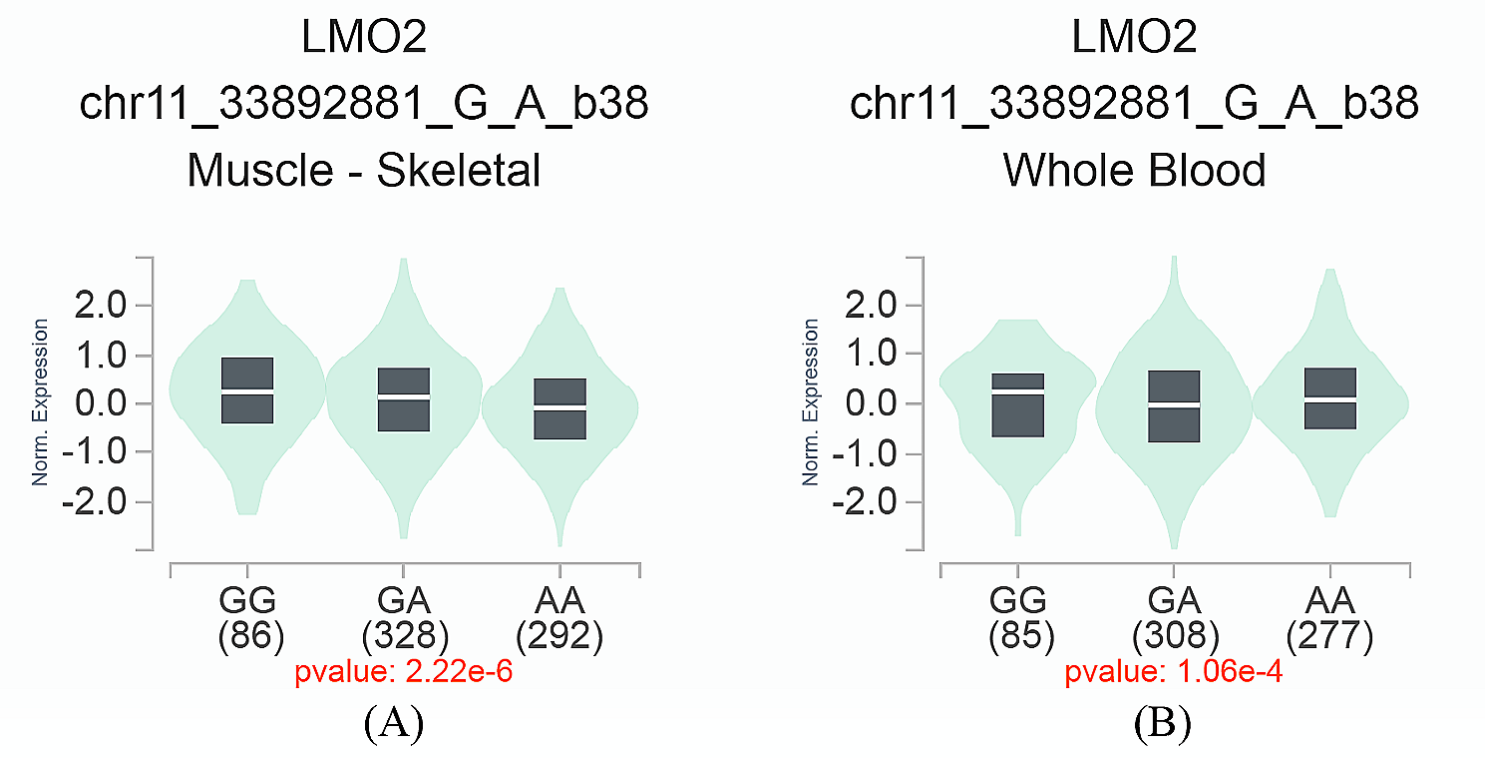
The eQTL analysis of the rs3758640 polymorphism on gene expression using data from GTEx database. In muscle-skeletal (**A**) and whole blood (**B**), compared to rs3758640 GG genotype, carriers with GA/AA genotypes were associated with reduced RNA expression of the *LMO2* gene

We further used the GTEx database to evaluate the effect of different genotypes of selected SNP on mRNA expression and the result was shown as in Fig. 1. As for rs3758640 A > G, compared to rs3758640 GG genotype, carriers with GA/AA genotypes were associated with reduced mRNA expression of the *LMO2* gene in muscle-skeletal and whole blood.

## Discussion

As one of the most frequently diagnosed childhood renal malignancies, high-risk Wilms tumor continues to threaten the health of patients with recurrent and unfavorable histological cases. Therefore, with the aim of identifying new biomarkers for early diagnosis and better targeted therapies, we performed a five-center case‒control study to explore the association of *LMO* family gene polymorphisms and Wilms tumor risk in a Chinese Han population for the first time. The results indicated that *LMO1* and *LMO2* genetic variants were associated with Wilms tumor susceptibility, which may help identify additional genetic susceptibility loci as novel biomarkers for the diagnosis and treatment of Wilms tumor.

The *LMO* family genes consist of four members, *LMO1-4*, each of which has been established to be responsible not only for many normal developmental processes but also for the initiation and development of various human cancers, including T-cell acute lymphoblastic leukemia, neuroblastoma, lung cancer, colorectal cancer, and breast cancer [21, 22, 25, 30–32]. The products of *LMO* family genes share a common two-tandem LIM domain structure, a highly conserved and cysteine-rich zinc binding motif without additional domains, mediating protein‒protein interactions instead of binding to DNA directly [33]. Thus, LMO proteins were previously regarded as adapter molecules for the formation of new versatile multiprotein complexes, which are crucial for participation in various cellular processes, including cell proliferation, cell self-renewal, differentiation, and metastasis [21, 34]. Interestingly, LMO proteins are capable of modulating transcriptional events by forming transcription factor regulators, thereby affecting target gene expression and cell fate decisions [21], suggesting a potential possible mechanism for the involvement of *LMO* family genes in the pathogenesis and progression of several cancers. Nevertheless, the exact details of the mechanism remain to be fully investigated.

Currently, among the *LMO* family genes, genetic associations between *LMO1* gene polymorphisms and tumor susceptibility have been most intensively studied. An increasing number of publications have identified that *LMO1* plays a determinant role in cancer susceptibility. In a previous GWAS performed in participants of European descent, Wang et al. identified that four SNPs (rs110419 A > G, rs4758051 G > A, rs10840002 A > G and rs204938 A > G) in the *LMO1* locus at 11p15.4 were strongly associated with the development of neuroblastoma, and subsequent replication studies successfully identified the same findings in different cohorts from the UK, USA and Italy [35]. Thereafter, the relationship between *LMO1* polymorphisms and neuroblastoma risk was further determined in other epidemiological case‒control studies among subject populations of various ethnicities [23, 30, 36, 37]. In addition to neuroblastoma, genetic variants in the *LMO1* gene also contribute to susceptibility to acute lymphoblastic leukemia (ALL). Beuten et al. discovered that the *LMO1* gene rs442264 A > G polymorphism significantly corresponded to an increased risk of developing ALL [38]. Considering the critical effect of *LMO1* gene polymorphisms on tumor risk, our team has spared no effort to investigate the relationship between *LMO1* gene polymorphisms and Wilms tumor susceptibility.

In 2017, in a case‒control study of 145 Wilms tumor patients and 531 cancer-free controls recruited from southern China, our team successfully genotyped four SNPs in the *LMO1* gene (rs110419 A > G, rs4758051 G > A, rs10840002 A > G and rs204938 A > G) and demonstrated for the first time that only rs110419 A > G had a protective effect against developing Wilms tumor [20]. Subsequently, we further explored the correlation between five other potentially functional *LMO1* gene polymorphisms (rs2168101 G > T, rs1042359 A > G, rs11041838 G > C, rs2071458 C > A, and rs3750952 G > C) and Wilms tumor risk among the same southern Chinese population mentioned above. The results suggested that only rs2168101 G > T could significantly decrease the risk of Wilms tumor, particularly in GT/TT genotype carriers. And the eQTL analyses showed that GT/TT genotypes reduced the expression level of *LMO1* gene in Muscle-Skeletal tissue compared to GG genotype. It is likely that rs2168101 G > T polymorphism participated in the development of Wilms tumor by regulating corresponding *LMO1* gene expression level [27]. However, the sample size was too small to limit the statistical power for the conclusion. Additionally, given the structural similarity of the *LMO* gene family, we reasonably speculated that *LMO2* and *LMO4* gene polymorphisms might be correlated to Wilms tumor susceptibility as *LMO1* gene did. Herein, we expanded the sample size to verify whether functional polymorphisms in the *LMO1* gene (rs2168101 and rs11603024), *LMO2* gene (rs2273799, rs3758640 and rs7933499) and *LMO4* gene (rs3766019) were associated with Wilms tumor susceptibility in a five-center case‒control study for Chinese population.

The current results implied that rs2168101 GT/TT genotypes in the *LMO1* gene were found to decrease the risk of developing Wilms tumor in the dominant model (adjusted OR = 0.74, 95% CI = 0.59–0.93, *P* = 0.009), which is consistent with the tendency for neuroblastoma and Wilms tumor in previous studies [27, 39, 40]. Oldridge et al. [39] observed that rs2168101 G > T is located in a super-enhancer of the first intron area within the *LMO1* gene. The rs2168101 G allele constituted a highly conserved transcription factor-binding site of GATA, which helped to promote the expression of the *LMO1* gene. When the rs2168101 G allele was mutated to the rs2168101 T allele, it abrogated the binding of GATA3 (*P* < 0.0001), thereby reducing the expression level of LMO1 [39]. This may account for the decreased risk of tumorigenesis for the rs2168101 G > T polymorphism, including Wilms tumor. When biologically functional SNP in *LMO1* gene was mutated, the expression of corresponding genes might be affected and was related to the occurrence of Wilms tumor. In contrast, in the recessive model (adjusted OR = 1.79, 95% CI = 1.25–2.57, *P* = 0.001), carriers with the *LMO1* rs2168101 TT genotype seemed to have an increased risk of Wilms tumor according to an adjusted OR of 1.79. This discovery was opposite to a meta-analysis publication that assessed the association between *LMO1* gene polymorphism and neuroblastoma risk. In that meta-analysis, the researcher found that the rs2168101 TT polymorphism was more likely to reduce the risk of neuroblastoma than the rs2168101 GT/GG polymorphisms, with an OR of 0.48 in the recessive model (TT vs. GT + GG: OR = 0.48, 95% CI = 0.31–0.75, *P* = 0.001) [41]. The obvious differences were mainly attributed to the following reasons: the relatively small size of subject populations, selection bias for participants, chance of the experiments, etc. As a result, replication studies are needed to further validate our findings. Moreover, another SNP locus in the *LMO1* gene, rs11603024 C > T, was detected to confer enhanced susceptibility to Wilms tumor in recessive model. The stratified analysis demonstrated that rs2168101 GT/TT genotypes played a protective role against the occurrence of Wilms tumor in the subgroups of age ≤ 18 months, males and clinical stages I/II compared to the rs2168101 GG genotype. Nevertheless, carriers with the rs11603024 TT genotype were significantly related to an increased risk of Wilms tumor than carriers with rs11603024 CC/CT genotypes among children aged over 18 months, females, males, clinical stages I/II and clinical stages III/IV.

Referring to the *LMO2* gene, only the rs7933499 G > A was significantly associated with an enhanced risk of developing Wilms tumor. From the stratified analysis, we found that carriers with the rs7933499 GA/AA genotypes could significantly increase the risk of Wilms tumor in the subgroup of age ≤ 18 and clinical stages I/II compared to subjects with the rs7933499 GG genotype. Regarding the *LMO4* gene, no significant association was observed between the rs3766019 G > A polymorphism and predisposition to Wilms tumor.

We further assessed the effect of different genotypes of selected SNPs on mRNA expression using the GTEx Portal. The eQTL analysis result of rs3758640 A > G showed that GA/AA genotype carriers were associated with reduced RNA expression of the *LMO2* gene in muscle-skeletal and whole blood compared to rs3758640 GG genotype. This implied that the different genotypes of rs3758640 may result in altered mRNA expression of the corresponding genes, which is associated with Wilms tumor susceptibility. However, other polymorphisms were not detected significant associations with Wilms tumor susceptibility. In fact, data resources of the GTEx Portal were almost from Caucasian populations, while our study was conducted in Chinese Han children. Therefore, it is possible that our results could not be exactly explained by the eQTL analysis. Further functional experiments are necessary to validate the current results, and to elucidate the potential pathogenesis of Wilms tumor in the future.

The strength of our study is that we enlarged the sample size from five independent hospitals, and we are the first to comprehensively examine the relationship between *LMO* family gene polymorphisms and Wilms tumor risk. We used Power and Sample Size Calculation software to evaluate statistical power. Assuming that the MAF of selected SNP was between 0.05 and 0.40, with an α level of 0.05, we were able to detect the risk of Wilms tumor with an OR value of 1.38–1.87 with 80% certainty. However, there are still several limitations for the current study that cannot be ignored. First, even if we expand the sample size, the size of the subject population seems to be relatively small, which inevitably weakens the statistical power. Meanwhile, the lack of combination analysis also weakens the strength of the conclusions. Second, due to the various genetic backgrounds for different ethnicities and regions, the conclusions obtained cannot be applied to other populations until they are validated by replicated studies. Third, the drawbacks of retrospective studies cannot be avoided, so some valuable information, such as environmental factors, was unavailable for further investigations. Finally, we only concentrate on the effect of several functional SNPs in the *LMO* family genes on Wilms tumor susceptibility, whereas there are many unknown potential polymorphisms in *LMO* family genes that deserve extensive study. Importantly, there is a strong need for functional experiments to further explore the underlying biological mechanisms of how highly related SNPs in the *LMO* family genes affect the risk of Wilms tumor.

## Conclusion

In brief, the current epidemiological genetic association study identified that several functional SNPs in the *LMO* family genes, namely, rs2168101 G > T and rs11603024 C > T in the *LMO1* gene and rs7933499 G > A in the *LMO2* gene, are significantly associated with susceptibility to Wilms tumor. Our findings are worth further validation in well-designed studies with large sample sizes among different races.

### Electronic supplementary material

Below is the link to the electronic supplementary material.

Supplementary Material 1

## Data Availability

No datasets were generated or analysed during the current study.

## Abbreviations

WT: Wilms tumor
WT1: Wilms tumor 1
GWAS: genome-wide association study
UTR: untranslated region
MAF: minor allele frequency
LD: linkage disequilibrium
TFBS: transcription factor binding site
HWE: Hardy-Weinberg equilibrium
OR: odds ratio
CI: confidence interval
GTEx: Genotype-Tissue Expression
eQTL: expression quantitative trait locus
ALL: acute lymphoblastic leukemia
