# Supplementary material for: LMO family gene polymorphisms and Wilms tumor susceptibility in Chinese children: a five-center case-control study
Source: BMC Cancer. 2024 Jun 27;24:772. doi: 10.1186/s12885-024-12557-3 (PMC11209997; doi:10.1186/s12885-024-12557-3)
Supplement: Supplementary file 1 — Supplementary Material 1 [file 12885_2024_12557_MOESM1_ESM.doc]

| **Table S1**. Frequency distribution of selected variables in Wilms tumor patients and cancer-free controls | | | | | |
| --- | --- | --- | --- | --- | --- |
| Variables | Cases (N=414) | | Controls (N=1199) | | *P* ^a^ |
|  | No. | % | No. | % |  |
| Age range, month | 1.00-148.63 | | 0.03-156.00 | | 0.118 |
| Mean ± SD | 31.14 ± 24.27 | | 32.31 ± 26.15 | |  |
| ≤18 | 143 | 34.54 | 466 | 38.87 |  |
| >18 | 271 | 65.46 | 733 | 61.13 |  |
| Gender |  |  |  |  | 0.218 |
| Female | 194 | 46.86 | 520 | 43.37 |  |
| Male | 220 | 53.14 | 679 | 56.63 |  |
| Clinical stages |  |  |  |  |  |
| I | 137 | 33.09 | / | / |  |
| II | 116 | 28.02 | / | / |  |
| III | 94 | 22.71 | / | / |  |
| IV | 49 | 11.84 | / | / |  |
| NA | 18 | 4.35 | / | / |  |
| SD, standard deviation; NA, not available.  ^a^ Two-sided χ^2^ test for distributions between Wilms tumor patients and cancer-free controls. | | | | | |

| **Table S2**. The potential functions of the selected polymorphisms from SNPinfo Web Server | | | | | | | |
| --- | --- | --- | --- | --- | --- | --- | --- |
| No. | rs | Chromosome | Position | Allele | TFBS | Splicing | miRNA |
| 1 | rs11603024 | 11 | 8242743 | C/T | Y | -- | -- |
| 2 | rs2273799 | 11 | 33870144 | C/T | Y | -- | -- |
| 3 | rs3758640 | 11 | 33871004 | A/G | Y | -- | -- |
| 4 | rs7933499 | 11 | 33859904 | A/G | -- | Y | Y |
| 5 | rs3766019 | 1 | 87583902 | G/A | -- | -- | -- |
| TFBS, transcription factor binding site. | | | | | | | |
